# Sulfatide interacts with and activates integrin αVβ3 in human hepatocellular carcinoma cells

**DOI:** 10.18632/oncotarget.9095

**Published:** 2016-04-29

**Authors:** Rong Wang, Bing Qi, Yi Wei Dong, Qian Qian Cai, Nian Hui Deng, Qi Chen, Chao Li, Yu Tong Jin, Xing Zhong Wu

**Affiliations:** ^1^ Department of Biochemistry and Molecular Biology, School of Basic Medical Sciences, Fudan University, Shanghai, P.R. China; ^2^ Key Laboratory of Glycoconjugate Research, Ministry of Public Health, Shanghai, P.R. China; ^3^ Yu Ying Children's Hospital, Wenzhou Medical University, Wenzhou, P.R. China

**Keywords:** integrin, sulfated cerebroside, clustering, signaling, hepatocellular carcinoma

## Abstract

Integrin αVβ3 is a malignant driver of anchorage-independence and tumor angiogenesis, but its dysregulation in hepatocellular carcinoma (HCC) remains unclear. In this study, we observed that sulfatide significantly promoted integrin αV(ITGAV) expression and wound closure in HCC. We also noted that elevated sulfatide profoundly stimulated integrin αVβ3 clustering and signaling. In the cells with integrin αVβ3 clustering induced by sulfatide, integrin β3 subunit was phosphorylated. Simultaneously, focal adhesion kinase (FAK), Src and paxillin were also phosphorylated. Treatment with FAK inhibitor resulted in robust suppression of FAK-Y397 and Src-Y416 phosphorylation stimulated by sulfatide, but not suppression of integrin β3 phosphorylation. Src inhibitors repressed Src-Y416 and FAK Y861 and Y925 phosphorylation, but not FAK-Y397 and integrin β3 phosphorylation. After mutation of integrin β3 (Y773F and Y785F), FAK or Src phosphorylation failed to be stimulated by sulfatide. Moreover, β3 Y773 and Y785 phosphorylation was suppressed by insulin-like growth factor receptor knockdown even in cells stimulated by sulfatide. In assays of immunoprecipitation and immunostaining with integrin αV or β3 antibody, labeled sulfatide was found in the complex and co-localized with integrin αVβ3. Taken together, this study demonstrated that elevated sulfatide bound to integrin αVβ3 and induced clustering and phosphorylation of αVβ3 instead of matrix ligand binding, triggering outside-in signaling.

## INTRODUCTION

Hepatocellular carcinoma (HCC) is one of the most malignant diseases worldwide, especially in Asia, where there is a high prevalence of chronic viral hepatitis infection. Tumor metastasis is the major cause of HCC-related mortality. Invasive primary HCC breaches the basement membrane and invades the lymphatic or blood microvessels, from where the tumor cells are transported to distant sites to form dormant micrometastases which may eventually acquire the ability of colonization and angiogenesis, enabling them to form a macroscopic metastasis [1]. HCC commonly metastasizes to the lungs, lymph nodes, adrenal gland and bone. Metastasis process is closely associated with cell migration and adhesion that are mostly integrin dependent, which resides on the cell surface and interacts with extracellular matrix (ECM) proteins. Although some integrins inhibit tumor cell proliferation via inducing apoptosis or cell death [2], Integrin αVβ3 is required for tumor angiogenesis [3] and anchorage-independent proliferation of cancer cells [4, 5]. Expression of integrin αVβ3 is upregulated in the vasculature associated with solid tumors [3, 4], indicating that integrin αVβ3 is involved in angiogenesis [6] and tumor metastasis [5, 7, 8]. Integrin αVβ3 is especially expressed on the most aggressive protruding tumor cells that invade normal tissue, in many cancers such as melanoma and carcinomas of pancreas [5]. The expression of integrin αVβ3 is often correlated with prostate cancer metastasis [9] and poor prognosis of patients with cervical carcinoma [10, 11]. Integrin αVβ3 expression contributes breast cancer cell migration and metastasis since exogenous expression of integrin αVβ3 in human breast cancer cells rescues the invasiveness and migration that are suppressed by MYC [7]. The antagonists of integrin αVβ3 obviously inhibited the tumor aggressiveness by inducing apoptosis of proliferative angiogenic vascular cells [4].

The noncatalytic cytoplasmic tail (CT) domain of integrin β3 subunit is laden with various phosphorylation sites, including two tyrosine residues, one serine, and multiple threonine residues [12]. The two tyrosine phosphorylation sites, which are located within NPXY(asparagine-proline-X-tyrosine) and/or NPXY-like motifs, can be recognized by talin or kindlin, an important integrin activator, and interact with phosphotyrosine binding domains of intracellular signaling mediators such as Dok1(docking protein 1) or Shc (Src homology and collagen homology) after phosphorylation, to regulate integrin activation and signaling [13]. In mice, the two tyrosine phosphorylation sites in the β3 subunit CT are Tyr747(Y747) and Tyr(Y759), which are involved in the signal transduction associated with tyrosine phosphorylation of focal adhesion kinase (FAK) and paxillin [14]. The corresponding tyrosine sites in humans are Y773 and Y785 [15]. Integrin tyrosine phosphorylation takes place upon ligand binding to the αIIbβ3 integrin. The cytoplasmic domain of integrin β3 subunit may serve as a substrate for v-Src kinase to regulate integrin activation and cell adhesion [16]. Blocking of αVβ3 binding to its ligands by adding exogenous polyamines to the fibronectin matrix can also prevent Src activation [17]. However overexpressed integrin αVβ3 recruits and activates c-Src by integrin β3 CT in independence on ligand binding, and forms an oncogenic unit to stimulate anchorage-independent proliferation of tumor cells [5]. Thus integrin αVβ3, the primary vitronectin receptor, plays an important role in the events of metastasis and angiogenesis. Any change of integrin αVβ3 activity will affect tumor proliferation and metastasis. However the dysregulation of integrin αVβ3 in tumor cells remains largely unknown and how overexpressed integrin αVβ3 activates c-Src without ligand binding is also not clear.

Sulfatide is a cell membrane component that is modified from cerebroside by adding a sulfate group by cerebroside sulfotransferase(CST). Sulfatide is significantly elevated in some cancers such as HCC. Nevertheless, the biological and pathological significance of elevated sulfatide in HCC is largely unknown [18]. Our previous studies showed that sulfatide promotes cell adhesion to vitronectin and metastasis of tumor cells in HCC [19] via modulation of integrin αV subunit expression by phosphorylated transcriptional factor Sp1(specificity protein 1) [20] and miR-223 [21]. However, the detailed process of how sulfatide stimulates integrin-mediated cell adhesion and migration, and how it triggers the phosphorylation cascades remain elusive. We hypothesize that sulfatide interacts with and activates the integrin αVβ3 heterodimer, and triggers signaling by phosphorylation cascades. To test this, we investigated the effects of elevated sulfatide on the clustering and phosphorylation of integrin αVβ3 and its signaling in HCC.

## RESULTS

### Sulfatide stimulation of HCC integrin αV expression

**Figure 1:**
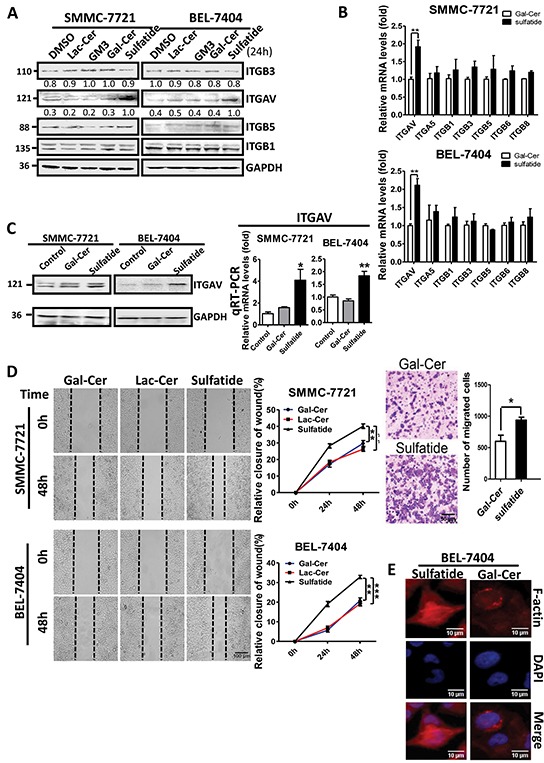
Sulfatide enhanced integrin αV expression and wound healing in HCC **A.** Protein levels of integrin αV, β1, β3 and β5 were measured by western blotting in SMMC-7721 and BEL-7404 cells treated with 2 μM Gal-Cer, sulfatide or an equivalent volume of DMSO (0.1% v/v) for 24 h. **B.** Expression of integrin subunits αV, α5, β1, β3, β5, β6 and β8 was examined by quantitative RT-PCR in HCC cells upon sulfatide treatment. **C.** Expression levels of integrin αV were examined by RT-PCR and western blotting. **D.** Representative micrograph of wound healing assays (left) in SMMC-7721 and BEL-7404 cells. The ratio of wound closure was measured after scratching and the quantitative analysis of relative closure is on the middle. Original magnification: 100×. Representative micrograph of cell migration assays in SMMC-7721 cells treated with Gal-Cer or sulfatide 24 h before the assay (right upper) and quantitative analysis. Original magnification: 100×. **E.** Representative images of actin filaments stained with rhodamine-conjugated phalloidin (red), and nuclei with DAPI (blue) in BEL-7404 cells treated with 2 μM sulfatide for 12 h. Original magnification: 400×. Bars indicate scales. Data are representative of three independent experiments. Gal-Cer: galactocerebroside, Lac-Cer: lactocerebroside, ** *P*<0.01, ***P<0.001.

As compared to DMSO control, galactocerebroside, lactocerebroside and ganglioside, sulfatide interestingly enhanced the expression of integrin αV subunit (Figure 1A) in both human hepatocellular carcinoma cells (SMMC-7721 and BEL-7404), but not integrin β1, 3 or 5. To investigate further the regulation by sulfatide, the expression profile of integrins was measured. The results of quantitative PCR showed that the level of integrin αV subunit expression was significantly elevated by sulfatide treatment in either SMMC-7721 or BEL-7404 cells (Figure 1B). The enhanced expression of integrin αV subunit by sulfatide was confirmed by western blotting and qPCR measurement (Figure 1C). We observed that exposure to sulfatide resulted in significantly faster wound closure in both SMMC-7721 and BEL-7404 cells than in the control cells (Figure 1D) by wound healing assay. SMMC-7721 cell migration through the microporous membrane was also significantly faster in the presence of sulfatide than in the control (Figure 1D right). Phallodin immunostaining of the cells treated with sulfatide indicated an increase in the amount of actin fiber polymerization in the vicinity of the leading plasma membrane compared to Gal-Cer (Figure 1E). The actin filaments were well organized in sulfatide-treated cells with more lamellipodia.

### Integrin αV clustering

**Figure 2:**
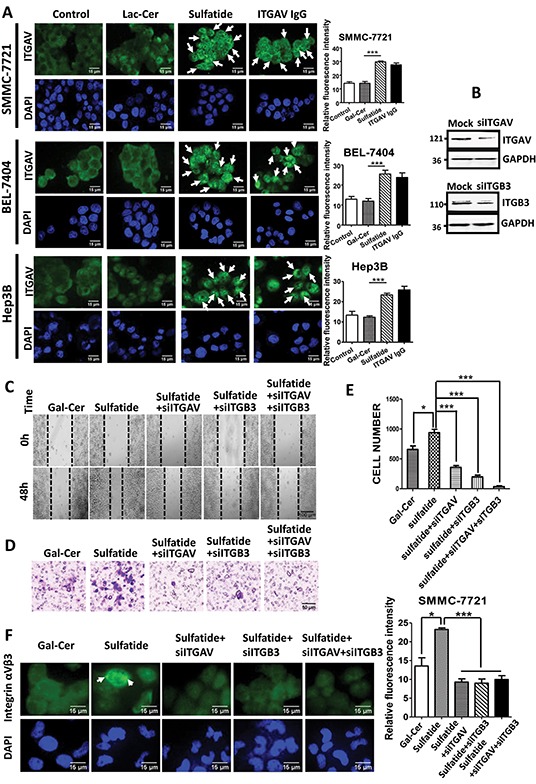
Sulfatide-induced clustering of integrin αVβ3 **A.** Representative images of integrin αV clustering in SMMC-7721, BEL-7404 and Hep3B cells treated with 2 μM sulfatide or 10 μg/mL integrin αV IgG antibody. Arrowheads indicate integrin clustering. Quantitative analysis of the clustering fluorescence signals is on the right. Original magnification: 400×. **B.** Knockdown of integrin αV or β3 expression (siITGAV and siITGB3) by RNA interference was validated via western blot analysis. **C.** Representative micrograph of wound healing in SMMC-7721 cells with integrin αV and β3 knockdown. **D.** Representative migration images of SMMC-7721 cells treated with sulfatide. **E.** Quantitative analysis of the migrated cells in **D, F.** Representative micrographs of integrin αVβ3 clustering in SMMC-7721 cells transfected with siITGAV, siITGB3, or co-transfected with siITGAV + siITGB3. Green: integrin αVβ3; Blue: nucleus. Original magnification: 400×. Data are representative of at least three independent experiments. *P<0.05, **P<0.01, ***P<0.001. n.s. not significant.

To uncover the initial regulation event in the HCC cells, we investigated and observed integrin clustering activity after treatment. Compared to control groups, cells treated with sulfatide showed extensive clustering of integrin αV, which was similar to the clustering induced by specific antibody against integrin αV (Figure 2A). In order to confirm the roles of integrin αVβ3 in sulfatide-mediated wound healing or cell migration in HCC, integrin αV and β3 expression was knocked down by RNA interference and confirmed by western blotting (Figure 2B) in SMMC-7721 cells in which other types of integrin were not targeted (data not shown). Knockdown of integrin αV or β3 subunit significantly suppressed sulfatide-stimulated wound closure (Figure 2C). Cell migration through the microporous membrane also significantly slowed down in the silenced cells (Figure 2D, 2E). Importantly, integrin αVβ3 clustering stimulated by sulfatide was completely suppressed by knockdown of integrin αV or β3 (Figure 2F).

### Activation of integrin αVβ3 signaling

**Figure 3:**
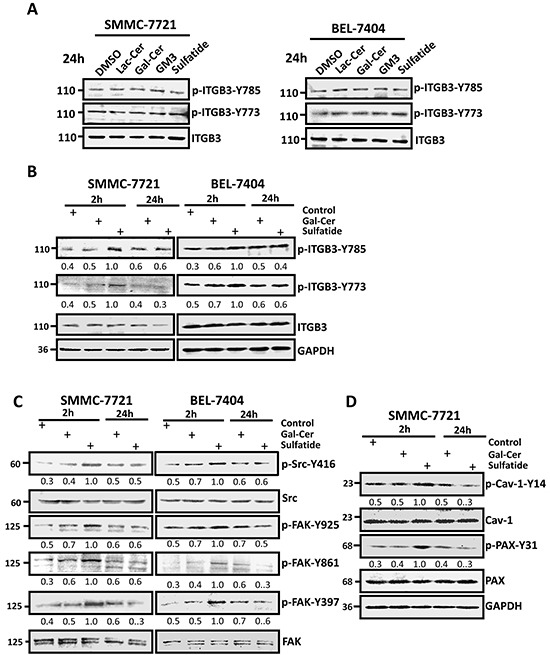
Sulfatide activated signaling in HCC cells **A.** and **B.** Phosphorylation levels of integrin β3-Y773 and β3-Y785 were measured by Western blotting 24 h or 2 h after treatment with 2 μM Lac-Cer, Gal-Cer, GM3, or sulfatide in SMMC-7721 and BEL-7404 cells. The bands were analyzed by densitometry and the relative value is shown under each band. **C.** Phosphorylation levels of FAK and Src were measured 2 or 24 h after sulfatide treatment by western blotting. The relative density value is shown under each band. **D.** Phosphorylation levels of paxillin and caveolin were measured 2 or 24 h after sulfatide treatment by western blotting. The relative value is shown under each band. Data are representative of three independent experiments.

Lateral association and clustering of integrins require integrin activation which is accompanied by tyrosine phosphorylation of the β3 subunit. Although the level of phosphorylated integrin β3 at Y773 and Y785 seemed not significantly different 24 h after sulfatide treatment (Figure 3A), a significant increase in the level of phospho-integrin β3-Y773 and phospho-β3-Y785 was observed within 2 h after treatment (Figure 3B). Simultaneously, shortly after SMMC-7721 and BEL-7404 cells were treated with sulfatide, the levels of the phosphorylated FAK-Y397, FAK-Y861, FAK-Y925, Src-Y416 were significantly strengthened (Figure 3C). The phosphorylated levels of paxillin-Y31 and caveolin-Y14 were also interestingly enhanced (Figure 3D). This suggested that sulfatide stimulated integrin αVβ3 and activated FAK-Src signaling.

### Sequential activation of FAK and Src

**Figure 4:**
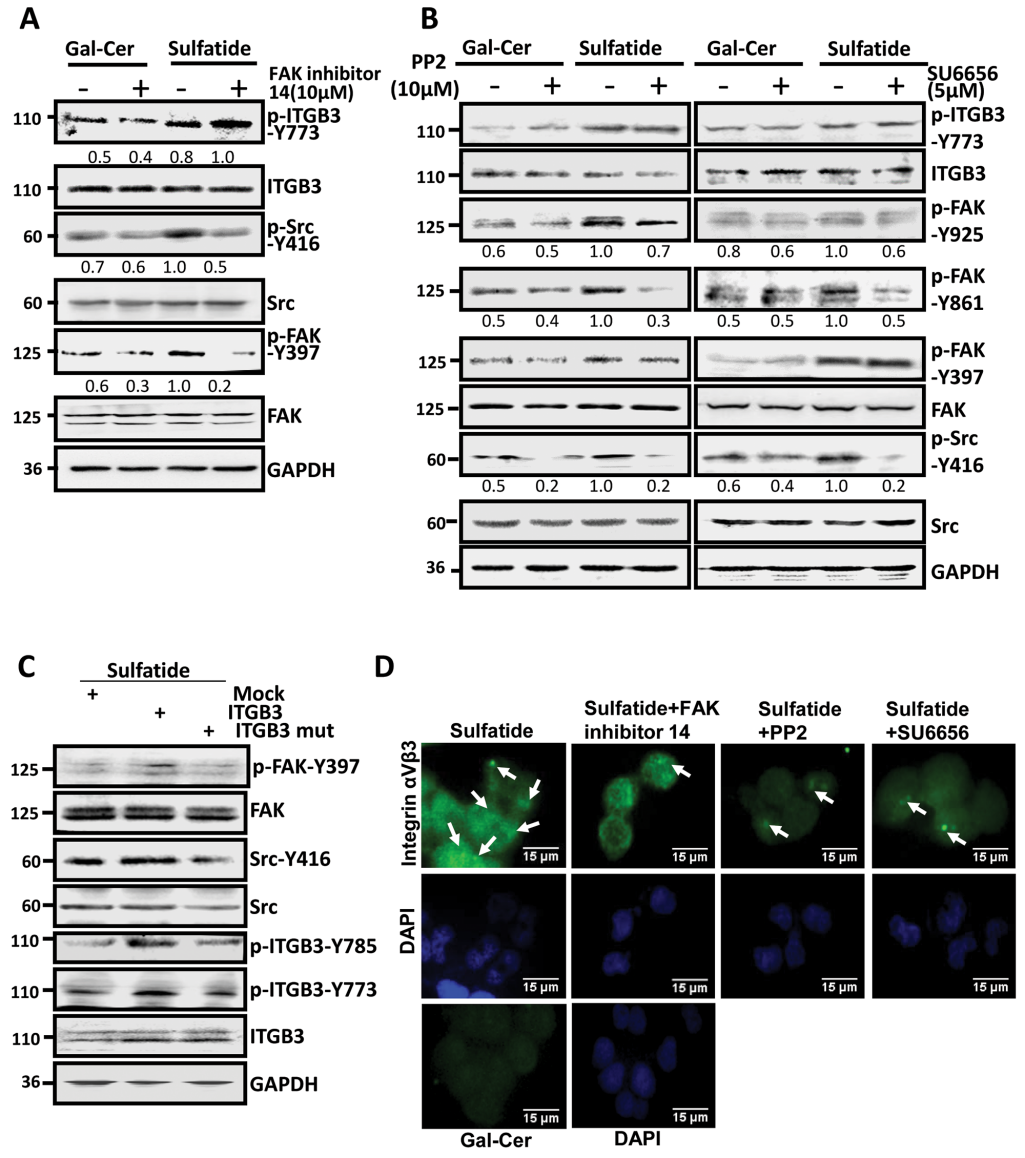
Sulfatide stimulated integrin αVβ3 and activated FAK-Src signaling **A.** Phosphorylation of Src, FAK-Y397 and integrin β3 subunit was measured in SMMC-7721 cells pretreated with FAK inhibitor 14 4 h prior to sulfatide. **B.** Phosphorylation levels of FAK-Y397, 861, 925 and integrin β3-Y773 were determined by western blotting in SMMC-7721 cells pretreated with PP2 or SU6656 4 h prior to sulfatide. **C.** Phosphorylation of FAK-Y397 and Src-Y416 induced by sulfatide was measured by western blotting in the cells transfected with mutated integrin β3 (Y773F or Y785F) construct. **D.** Representative micrographs are integrin αVβ3 clustering induced by sulfatide after FAK and Src inhibition in SMMC-7721 cells. Green: integrin αVβ3; Blue: nucleus. Original magnification: 400×. Data are representative of three independent experiments.

Now in the cells treated with sulfatide, integrin αVβ3 was activated. FAK and Src were also observed that they were phosphorylated and activated. To study further their role in the integrin activation and signaling, we examined both FAK and Src inhibitors, and observed that they were phosphorylated and activated sequentially. FAK inhibitor 14 significantly suppressed the sulfatide-stimulated phosphorylation of FAK on Y397 and attenuated the activation of Src (p-Src-Y416), but not phosphorylated integrin β3 (β3-Y773) (Figure 4A). However, Src inhibitors, PP2 and SU6656, decreased significantly sulfatide-stimulated FAK phosphorylation of p-FAK-861 and p-FAK-Y925, but not p-FAK-Y397 and p-integrin β3-Y773 (Figure 4B). These suggested that Src-Y416 phosphorylation was after FAK-Y397, but before FAK-861 and FAK-Y925 phosphorylation. We then mutated integrin β3 Tyr773 and Tyr785 to phenylalanine, and found that the mutation of integrin β3 markedly decreased FAK Y397 and Src Y416 phosphorylation in the sulfatide-stimulated cells (Figure 4C). However, sulfatide-stimulated integrin αVβ3 clustering was attenuated by FAK inhibitor 14 and Src inhibitors (PP2 and SU6656) (Figure 4D). Also, sulfatide-stimulated wound healing was significantly inhibited by Src inhibitors (data not shown). Thus, sulfatide stimulated integrin αVβ3 clustering and phosphorylation, which triggered FAK-Src signaling, leading to HCC cell migration.

### Sulfatide binds to integrin αVβ3

**Figure 5:**
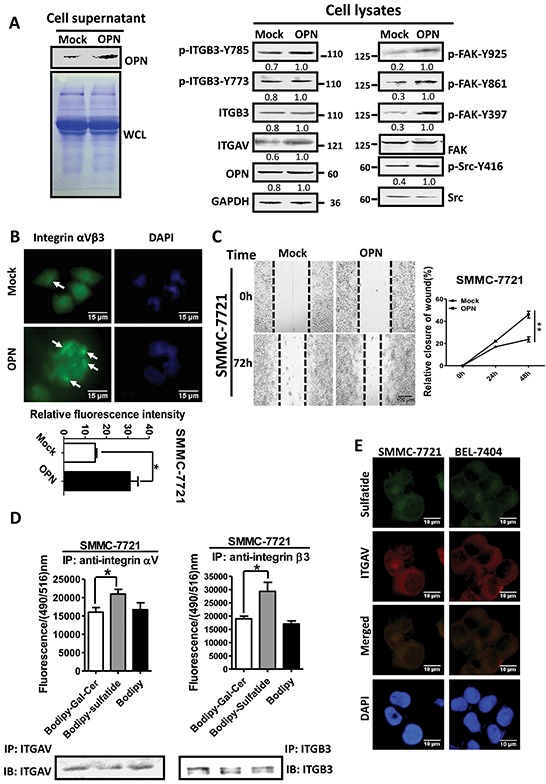
Interaction of sulfatide with integrin **A.** Overexpression of OPN was validated by western blotting in the culture medium of SMMC-7721 cells after transfection (left). Integrin αV, β3, FAK and Src were measured by western blotting in cells overexpressing OPN (right). CBB: Coomassie brilliant blue staining. **B.** Representative images of integrin αVβ3 clustering in OPN-overexpressing SMMC-7721 cells. Quantitative analysis is shown on lower panel. Green represents integrin αVβ3 and blue indicates nucleus. Original magnification: 400×. **C.** Representative micrograph of wound healing assays in SMMC-7721 with OPN overexpression. The relative wound closure was quantitatively analyzed. **D.** Fluorescence signal was measured at a wavelength of 516 nm excited at 490 nm (Upper) after incubation with Bodipy-labeled sulfatide and immunoprecipitation by antibody against integrin αV or β3. The integrin proteins were determined via western blot analysis in the immunoprecipitated complex (Lower). **E.** Representative images of immunofluorescent staining of sulfatide and integrin αV in SMMC-7721 and BEL-7404 cells. Co-localization, as indicated in Merged, is yellow. Original magnification: 400×. Data were representative of at least three independent experiments. *P<0.05, **P<0.01.

The integrin clustering correlates with integrin activation, which needs ligand binding in the process since the activated integrins require ligands to form integrin clusters and to induce tyrosine phosphorylation of the β subunit. OPN is a small integrin-binding protein and can bind to integrin αV via its RGD motif [22]. To understand the regulation of integrin by sulfatide, we examined the effect of OPN in HCC cells on integrin clustering. The results showed that overexpression of OPN indeed induced clustering of integrin αVβ3 and phosphorylation of FAK and Src (Figure 5A, 5B). Meanwhile, OPN also enhanced the wound closure capacity of SMMC-7721 cells (Figure 5C). The effect of OPN that bound integrin αVβ3 on HCC seemed similar to sulfatide-stimulated HCC cell migration. To examine if sulfatide interacted with integrin αVβ3, SMMC-7721 cells were incubated with 10 μM fluorescent BODIPY-tagged sulfatide or BODIPY-tagged Gal-Cer. The lysate was immunoprecipitated with integrin αV or β3 antibody. Fluorescence intensity was measured in the pellets precipitated by specific integrin αV or β3 antibody and the fluorescence signal was significantly higher in the Bodipy-sulfatide group than in the Bodipy or Bodipy-Gal-Cer control (Figure 5D). Sulfatide was also co-localized with integrin αV in fluorescence immunostaining (Figure 5E). These results suggested that sulfatide interacted with integrin αVβ3.

### RGD peptide prevents sulfatide-stimulated signaling and cell migration

**Figure 6:**
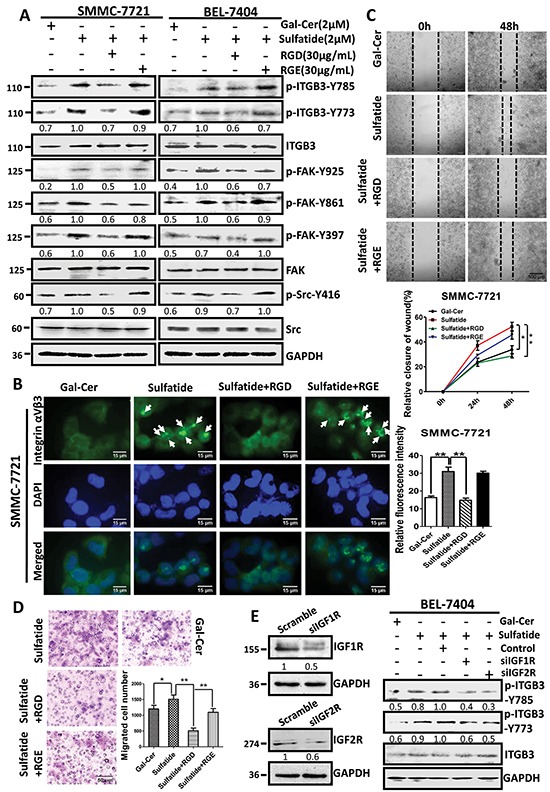
Interaction of sulfatide with integrin involved RGD **A.** Phosphorylation of integrin β3, Src and FAK was measured by western blotting in SMMC-7721 and BEL-7404 cells pretreated with RGD or RGE peptide 4 h prior to sulfatide. **B.** Representative images of the integrin αVβ3 clustering in SMMC-7721 cells pretreated with 30 μg/mL RGD or RGE 4 h prior to sulfatide. Green: integrin αVβ3; Blue: nucleus. Original magnification: 400×. **C.** Representative images of wound closureand quantitative analysis. Original magnification: 100×; bars: SD. **D.** Representative micrograph of migration in SMMC-7721 cells pretreated with 30 μg/mL RGD prior to sulfatide and quantitative analysis. **E.** Knockdown of IGFR expression (siIGF1, 2R) and the phosphorylation of integrin β3 in SMMC-7721 cells. Knockdown of IGF1R or IGF2R was validated by western blotting (left panel). Levels of phosphorylated integrin β3-Y773 and β3-Y785 were measured in the cells with IGF1R or IGF2R knockdown after sulfatide treatment (right panel). Data were representative of at least three independent experiments. Scramble: control plasmid, *P<0.05, **P<0.01.

Integrin αVβ3 binding to ligand molecules is through RGD-binding sites, and upon ligand binding, the cytoplasmic domain of the integrin β subunit associates with signaling and cytoskeletal proteins. To investigate the role of RGD-binding site in αVβ3 and sulfatide interaction, we used the linear synthetic RGD peptide (GRGDSP) to block the RGD binding site of integrin αVβ3 with the negative control GRGESP. RGD peptide inhibited sulfatide-stimulated phosphorylation of integrin β3-Y773, β3-Y785, FAK-Y397, FAK-Y861, FAK-Y925 and Src-Y416 in both SMMC-7721 and BEL-7404 cells (Figure 6A). To determine further the impact of RGD peptide on sulfatide-induced integrin clustering and cell migration, we performed immunofluorescence and migration assays. RGD peptide significantly suppressed the clustering of integrin αVβ3 stimulated by sulfatide, whereas RGE peptide did not (Figure 6B). RGD peptide significantly retarded the wound closure capacity of SMMC-7721 cells even in the presence of sulfatide (Figure 6C). The migration was also slowed down (Figure 6D). This suggested that RGD-binding site was important in sulfatide interaction with integrin αVβ3, sulfatide-induced FAK-Src signaling, and cell behaviors.

### IGFR is important in phosphorylation of integrin β3

In sulfatide-induced activation of integrin β3/FAK/Src signaling, integrin β3 was phosphorylated. However, both integrin β3 and sulfatide lack kinase activity. The phosphorylation of integrin β3 subunit needs an additional kinase molecule. In this process of sulfatide-stimulated activation of integrin αVβ3, the inhibitors of Src kinase failed to prevent the phosphorylation of β3 subunit. We silenced IGF1R and IGF2R to investigate their roles in the phosphorylation of integrin αVβ3 and the results indicated that the sulfatide-stimulated phosphorylation of integrin β3 was suppressed in the cells with silenced IGF1R or IGF2R expression (siIGF1R and siIGF2R) (Figure 6E). Therefore, IGFRs were important in the phosphorylation of integrin β3 subunit Tyr773 and Tyr785 induced by sulfatide.

## DISCUSSION

Integrin αV abnormality is associated with malignancy in many cancers. The tissues of non-small cell lung cancer were observed abnormal elevation of integrin αV expression that was believed to promote tumor proliferation and progression [23]. Overexpression of integrin αV correlates well with the shorter survival of patients with colorectal cancer [24]. Integrin αVβ3 mediates the interaction with extracellular matrix as an adhesion molecule, but overexpressed integrin αVβ3 initiates endothelial cell survival in anchorage independence and tumor metastasis without ECM ligand binding [5]. Integrin αVβ3 is also considered as the malignant driver of tumor cell stemness [10, 25]. Thus, activation and clustering of integrin αVβ3 is important for tumor cell invasion, migration and metastasis. The present study showed that sulfatide significantly induced integrin αVβ3 clustering and stimulated phosphorylation of integrin β3 intracellular tail on Tyr773 and Tyr785. The phosphorylated intracellular domain of integrin β3 subunit is important for mediating outside-in signaling since it triggers the signaling cascade [12]. We also observed that FAK-Y397 and Src-Y416 were phosphorylated and activated sequentially. FAK-Y397 was phosphorylated earlier than Src-Y416, since Src inhibitor did not suppress FAK-Y397 phosphorylation, and FAK inhibitor suppressed Src-Y416 phosphorylation even under sulfatide stimulation. Subsequent phosphorylation of FAK-Y861 and FAK-Y925 was caused by Src activation since Src inhibitor also suppressed them. Paxillin, a scaffold protein, was phosphorylated after sulfatide treatment. This suggests that the signal sequence was similar to outside-in pattern. The phosphorylated tyrosine of paxillin was temporally and spatially regulated to provide additional binding sites for recruitment of signaling proteins to the plasma membrane, and to contribute to organization of the actin cytoskeleton at sites of focal adhesion [26]. Caveolin, which drives the formation of plasma membrane caveolae and anchors them to the actin cytoskeleton, was also phosphorylated. Phosphorylated caveolin-1 is implicated in mediation of focal adhesion turnover and integrin-regulated membrane domain internalization [27]. The rapid exchange of focal adhesion and internalization contributed to the cell migration of HCC stimulated by sulfatide, especially at the rear of the cells.

Tyrosine phosphorylation of integrin β1 subunit inhibits talin and kindlin binding to NPXY sequence leaving room for other proteins to bind, for example, filamin and tensin [28]. Loss of talin and kindlin from the CT domain of integrin β3 leads to inactivation of the integrin, which is conducive to recycling of integrin molecules and is important for persistent cell migration. The integrin β2 subunit has tyrosine at 735 and mutation of this residue impairs integrin recycling [29] and cell migration. Integrin αVβ3 clustered upon sulfatide stimulation and β3 subunit was phosphorylated. The phosphorylated integrin β3 subunit provided a binding site for FAK-Y397 autophosphorylation. However, integrins have no kinase activity for themselves autophosphorylation, and phosphorylation of integrin β3 subunit needs additional molecular involvement. The phosphorylation of threonine or serine on the cytoplasmic domain of β subunit is related to protein kinase C and the small G protein signaling pathway. However, it is not clear and controversial for the tyrosine phosphorylation [30] located within NPXY and/or NPXY-like motifs, although association and interaction were noted between integrin β3 CT and Src kinase [31]. Integrin β3 phosphorylation by Src kinase should be reasonable in inside-out signaling of integrins. However, clustered integrin αVβ3 can mediate cross-activation of Src kinase that is in the complex to stimulate cell adhesion and migration upon ligand binding [17, 31], which fits outside-in signaling. In the current study, we observed that activation of Src was after FAK phosphorylation, which was similar to outside-in signaling of integrins, and interestingly noted the role of IGFR in the phosphorylation of integrin β3 because sulfatide failed to stimulate integrin β3 phosphorylation after silencing of IGF1R and IGF2R, suggesting integrin β3 phosphorylation was associated with IGFR. This is consistent with our previous studies in which IGF1R was a functional target of miR-223 [32], and miR-223 expression is down-regulated by sulfatide in HCC [21]. Hence, sulfatide enhanced phosphorylation of integrin β3 through regulation of IGF1R and miR-223. Additionally, IGFR can be physically linked to integrin αVβ3 in HCC [33], and formation of the complex αVβ3–IGF1R does not require IGF1R activation and β3 phosphorylation [34]. Exogenous IGF stimulated β3 (Y747) phosphorylation, although Src was blocked by Src inhibitor PP2, not depending on anchorage.

How sulfatide induces integrin αVβ3 phosphorylation by IGFR after stimulation should be the next question. We then investigated the interaction between sulfatide and integrin αVβ3, and observed that sulfatide bound to integrin αVβ3 via the RGD binding domain. Blocking this domain by RGD peptide suppressed sulfatide binding and integrin αVβ3 clustering or phosphorylation. Therefore, it was the sulfatide binding that induced clustering and phosphorylation of integrin αVβ3. Integrin clustering classically requires the interaction with and binding of ECM ligands to induce activation of integrin. As integrin binds to the ECM ligand, it forms the first contact with the ECM in the lamellipodia, and the integrin activator, talin or kindlin associates with the cytoplasmic domain of the β subunit to induce conformational change and integrin activation. This process is followed by integrin lateral movement on the membrane, clustering, and phosphorylation of β subunit. Sulfatide, as an important component of the cell membrane, is often elevated in HCC [18]. The elevated sulfatide stimulated integrin αV expression. The increased integrin αV formed αVβ3 with β3 integrin and interacted with sulfatide, leading to αVβ3 clustering and β3 cytoplasmic tail phosphorylation that provided a binding site for FAK autophosphorylation. Src was then activated and acted as an oncogenic driver to stimulate proliferation in anchorage-independence. Therefore interaction of sulfatide with integrin αVβ3 substituted ECM ligand binding to integrin αVβ3 and induced integrin αVβ3 clustering and phosphorylation which sent signals to FAK-Src pathway to accelerate cell growth in HCC independent on ECM ligand binding.

## MATERIALS AND METHODS

### Materials

Rabbit antibodies against phosphorylated FAK (pFAK, Tyr397, Tyr861 and Tyr925), phospho-paxillin-Y31, phospho-caveolin-1-Y14, phosphorylated integrin β3 (Tyr773/Tyr785) and non-phosphorylated FAK, paxillin and caveolin-1 were from Bioworld Technology (St. Louis Park, MN, USA). Phosphorylated FAK Tyr407 was from Abcam (Cambridge, UK). Phospho-Src-Y416 was from Cell Signaling Technology (Danvers, MA, USA). Antibodies against integrin αV, β3, Src and FAK inhibitor 14 were from Santa Cruz Biotechnology (Santa Cruz, CA, USA). Integrin αVβ3 antibody LM609 was from Millipore (Temecula, CA, USA). Integrin β1 and β5 antibodies, PP2(1H-Pyrazolo[3,4-d]pyrimidin-4-amine, 3-(4-chlorophenyl)-1-(1,1-dimethylethyl)-) and SU6656((Z)-N, N-dimethyl-2-oxo-3-((4,5,6,7-tetrahydro-1H-indol-2-yl)methylene)indoline-5-sulfonamide) were from Merck (Darmstadt, Germany). Sulfatide, galactocerebroside, lactocerebroside and ganglioside (GM3) were from Sigma–Aldrich (St. Louis, MO, USA). Peptides GRGDSP and GRGESP with 99% purity were provided by China Peptides Co. (Shuzhou, China). Bodipy FL-C5 was from Molecular Probes (Carlsbad, CA, USA).

### Cell culture

HCC cell lines SMMC-7721 and BEL-7404 were obtained from Shanghai Institute of Biochemistry and Cellular Biology, Chinese Academy of Science, and authenticated. Cells were cultured in Dulbecco's Modified Eagle's Medium (DMEM; Gibco, NY, USA) complete medium containing 10% newborn calf serum. For treatment, cells were cultured in DMEM containing 2 μM sulfatide, galactocerebroside, or lactocerebroside from stock solution in DMSO. An equal amount of DMSO (0.1% v/v) was added to the control group.

### Plasmid construction

The short hairpin sequences, 5′-GATCCGCTGAGCTCATCGTTTCCATTCAAGAGATGGAAACGATGAGCTCAGCAGA-3′ and 5′-AGCTTCTGCTGAGCTCATCGTTTCCATCTCTTGAATGGAAACGATGAGCTCAGCG-3′, which specifically targeted the mRNAs of integrin αV subunit, 5′-GATCCCCAAGACTCATATAGCATTTTCAAGAGAAATGCTATATGAGTCTTGGAGA-3′ and 5′-AGCTTCTCCAAGACTCATATAGCATTTCTCTTGAAAATGCTATATGAGTCTTGGG-3′ for targeting integrin β3 subunit, 5′-GATCCGCGGCTGGAAACTCTTCTATTCAAGAGATAGAAGAGTTTCCAGCCGCAGA-3′ and 5′-AGCTTCTGCGGCTGGAAACTCTTCTATCTCTTG AATAGAAGAGTTTCCAGCCGCG-3′ for insulin-like growth factor 1 receptor (IGF1R), 5′-GATCCCCTGGGAACTCCTGAATTTTTCAAGAGAAAATTCAGGAGT TCCCAGGAGA-3′ and 5′-AGCTTCTCCTGGGAACTC CTGAATTTTCTCTTGAAAAATTCAGGAGTTCCCAG GG-3′ for IGF2 receptor (IGF2R) were designed through the website of Life Technologies (http://rnaidesigner.lifetechnologies.com/rnaiexpress/design.do). Synthetic 55-bp oligonucleotides containing the siRNA sequences were annealed and ligated to pSilencer 4.1 vector. Tyrosine to phenylalanine mutations of integrin β3 at 773 and 785 were introduced into the integrin β3 cDNA by PCR mutagenesis using primers consisting of 5′-AAAGAATTCATGCGAGCGCGGCC-3′, 5′-TGGCT TCTTTAAACAGTGGGT-3′, 5′-CAACAACCCACTGT TTAAAGAGG-3′, and 5′-GGGTCTAGATTAAGTGCCCCGGAACGTGAT-3′.

### Western blotting

After cells were harvested and lysed with 1% SDS containing 50 mM NaF, 1.5 mM Na_3_VO_4_, and 0.5 mM phenylmethanesulfonyl fluoride (PMSF). The protein was resolved on 10% SDS-polyacrylamide gels and electrophoretically transferred to a polyvinylidene fluoride membrane. Blocked with 8% milk for 2 h at room temperature, membranes were incubated overnight with primary antibodies. After washing, the blots were incubated with horseradish-peroxidase-conjugated secondary antibody, and protein bands were visualized using enhanced chemiluminescence detection kit and captured by an E/M camera. The density of protein bands was analyzed with Band Analyzer software(Tenon, Shanghai, China).

### Clustering and immunofluorescence

The cells were incubated with 2 μM Gal-Cer, sulfatide or 10 μg/ml anti-integrin αVβ3 antibody for 1 h at 10°C in DMEM. Cells were fixed with freshly prepared 4% paraformaldehyde in PBS for 20 min at room temperature, and blocked with 1% bovine serum albumin in PBS for 2 h at room temperature without permeabilization. Cells were incubated with primary antibodies against integrin αV or αVβ3 overnight at 4°C, followed by FITC-conjugated secondary antibody and DAPI (4′,6-diamidino-2-phenylindole) at room temperature in the dark for 2 h. The coverslips were mounted on glass slides. Images were captured using an Olympus fluorescent microscope (Tokyo, Japan).

### F-actin staining

Before fixing, cells in a Lab-Tek chamber (Nunc, Naperville, IL) were treated with 2 μM Gal-Cer or sulfatide for 48 h at 37°C. After washing with PBS four times and permeabilization with 0.1% Triton-X-100 in PBS in a shaker for 5 min, the cells on slides were incubated with rhodamine–phalloidin (1 μg/mL) and DAPI for 20 min at room temperature. Then slides were rinsed in PBS and mounted for microscopy. Images were captured using an Olympus fluorescent microscope.

### Wound healing assay

The cells were seeded at a density of 5.0×10^5^ cells into each well of six-well plates. The cell monolayers were scratched with a sterile micropipette tip, washed with PBS to remove the detached cells, and incubated with 2 μM sulfatide and Gal-Cer. The scratches were monitored every 24 h until they closed. The rate of wound closure was determined by calculating the percentage of the area covered by migrating cells in the initial wound. All experiments were repeated three times independently.

### Cell migration assay

Cell suspensions (100 μL) with 2 μM Gal-Cer or sulfatide were seeded in Millicell inserts (Millipore, Billerica, MA, USA) with polyethylene terephthalate microporous membrane (8 μm pore size) at a density of 2×10^5^/mL in serum-free DMEM in triplicate, and medium containing 10% fetal bovine serum was added to the lower chamber. Cells were allowed to migrate across polyporous membrane for 12 h at 37°C. The migrated cells on the downside of the membrane were fixed with methanol, stained with 0.5% crystal violet, and counted. The average number of migrated cells was counted from three independent experiments. Each set of experiments was performed in triplicate separately.

### Bodipy labeling and immunoprecipitation

Bodipy labeling assay was performed as described previously [35]. Sulfatide (0.2 mg) reacted with 0.2 mg Bodipy FL-C5 at room temperature for 1 h with shaking. After the reaction, labeled sulfatide was separated by thin-layer chromatography and dissolved in DMSO. The purified Bodipy–sulfatide was incubated with SMMC-7721 cell lysate in buffer containing 0.5% Nonidet P-40, 120 mM NaCl, 50 mM Tris–HCl pH 8.0, 1 mM Na_3_VO_4_, 10 μg/mL aprotinin, 2 mM PMSF and 10 mM NaF for 1 h at 37°C. The lysates were immunoprecipitated with anti-integrin αV or β3 antibody along with protein A/G-agarose beads. Immunocomplex-captured beads were washed with lysis buffer. Bodipy fluorescence was determined (excitation, 490 nm; emission, 516 nm). Afterwards, immunocomplexes captured by beads were boiled in Laemmli sample buffer. The proteins were separated by 10% SDS-PAGE and western blotted using anti-integrin αV or β3 antibody.

### Statistical analysis

Data are presented as means ± SD. Statistical differences were measured using one-way ANOVA and Student's *t* test. Statistical significance was set at *P* < 0.05 or *P* < 0.01.
